# A rare case of idiopathic cholestasis: Clinical conundrums complicating enzalutamide therapy in metastatic prostate cancer

**DOI:** 10.1002/ccr3.2427

**Published:** 2019-09-26

**Authors:** Jun Liu, George Agyapong, Debashish Misra, C. Douglas Taylor, David A. Hirsh

**Affiliations:** ^1^ Harvard Medical School Boston MA USA; ^2^ Department of Medicine Cambridge Health Alliance Cambridge MA USA

**Keywords:** adverse drug reactions, cholestasis, enzalutamide, paraneoplastic syndrome, prostate cancer

## Abstract

Current safety data affirms enzalutamide does not cause clinically significant liver dysfunction that warrant therapy cessation. Therefore, clinicians should not withhold potentially successful therapy merely for suspected hepatotoxicity or PnC.

## Abstract 2

We report a case of paraneoplastic cholestasis (PnC) coinciding with enzalutamide initiation and masquerading as an enzalutamide‐related adverse event. Current safety data affirms that enzalutamide does not cause clinically significant liver dysfunction. Therefore, clinicians should not withhold potentially successful therapy merely for suspected hepatotoxicity or PnC.

## Graphical abstract

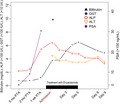

## 1 BACKGROUND

 Prostate cancer (PCa) rarely manifests with paraneoplastic cholestasis (PnC), a nonmetastatic liver dysfunction that occurs in malignancy without direct hepatobiliary obstruction or hepatic infiltration 1, 2, 3, 4, 5. Uncommon and underrecognized entities like PnC hold potential implications for clinical management. 

Enzalutamide was approved in 2012 for metastatic castration‐resistant prostate cancer (mCRPC) based on its superior survival benefits and favorable safety profile.^6^, ^7^, ^8^, ^9^ Here we report a case of idiopathic cholestasis in a patient who had just initiated enzalutamide therapy for mCRPC. We highlight clinical conundrums that influenced our approach to distinguishing possible enzalutamide‐related hepatotoxicity from PnC, emphasizing patient‐centered perspectives for managing underlying PCa in this and similar contexts.

## 2 CASE DESCRIPTION

**Figure 1:**
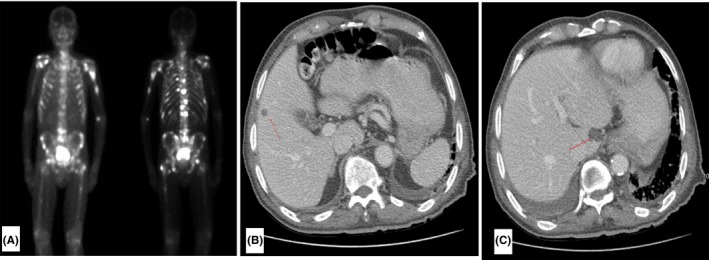
Skeletal metastases on bone scan (A) and no hepatobiliary metastases on contrast CT (B, C, arrows show benign cystic lesions in the liver) at the time of initial PCa diagnosis

**Figure 2:**
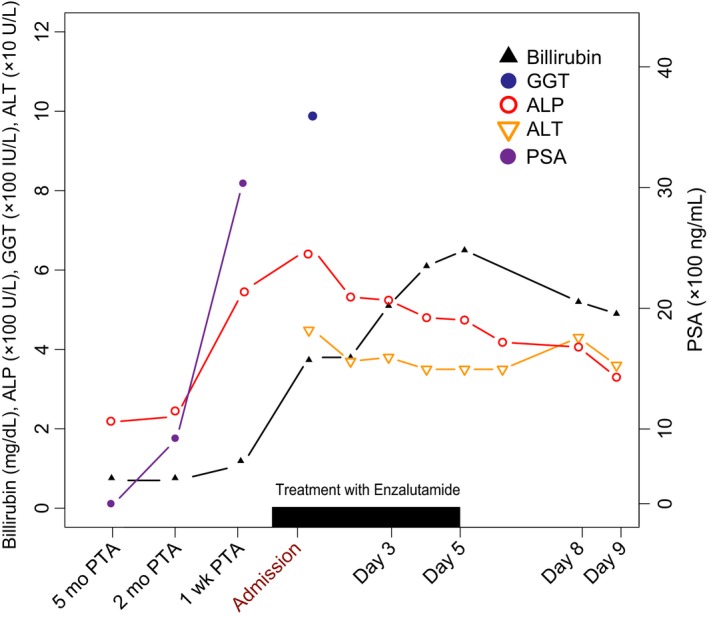
Precipitous PSA elevation prior to admission along with trends in elevated bilirubin, alkaline phosphatase (ALP), gamma‐glutamyl transferase (GGT, on admission), and alanine aminotransferase (ALT). Enzalutamide therapy continued through Day 5 of admission. It was initially discontinued given initial concern for drug induced‐toxicity; and not resumed per patient's wishes

An 88‐year‐old man with a history of metastatic PCa and newly diagnosed stage III chronic kidney disease (CKD) was admitted with one week of severe weakness, anorexia, and jaundice. The patient was diagnosed with PCa eight months prior to admission (PTA). He had an elevated prostate‐specific antigen (PSA) of 4280 ng/mL and marked prostate enlargement on CT scan, but had declined prostate biopsy for tumor staging at that time. Additional imaging also showed advanced disease with skeletal metastases (Figure 1A, bone scan) and visceral involvement in the lung (not shown). He had no liver metastases on contrast CT during initial diagnostic workup (Figure 1B, taken prior to onset of CKD). Following the diagnosis, the patient started bicalutamide and leuprolide androgen‐deprivation therapy. PSA level decreased to a minimum of 357 ng/mL after 1 month of treatment. However, subsequent testing showed disease progression with rapid doubling of PSA from 357 ng/mL (5 months PTA) to 919 ng/mL (2 months PTA), despite endocrine therapy adherence (Figure 2). His disease progressed precipitously 1 week PTA, with a markedly elevated PSA at 3030 ng/mL (Figure 2). He had started enzalutamide for mCRPC just prior to his admission. Bicalutamide was discontinued then without other medication changes. His other medications included leuprolide, finasteride, doxazosin, metoprolol, omeprazole, ferrous gluconate, multivitamin, psyllium, and a nutritional supplement (Table 1). The patient reported no history of blood transfusions, recent travel, or use of alcohol, tobacco, or drugs. He had no personal or family history of hepatobiliary disease.

**Table 1:** List of medications and supplements that the patient was taking prior to admission

Medication	Dosage
Enzalutamide	160 mg/d
Finasteride	5 mg/d
Leuprolide	22.5 mg injection every 3 mo
Multivitamin	1 tablet/d
Ensure liquid (nutritional supplement)	24 Oz/d
Doxazosin	50 mg/d
Ferrous gluconate	324 mg/d
Metoprolol	100 mg/d
Omeprazole	20 mg/d
Psyllium	0.52 mg twice/d

**Figure 3:**
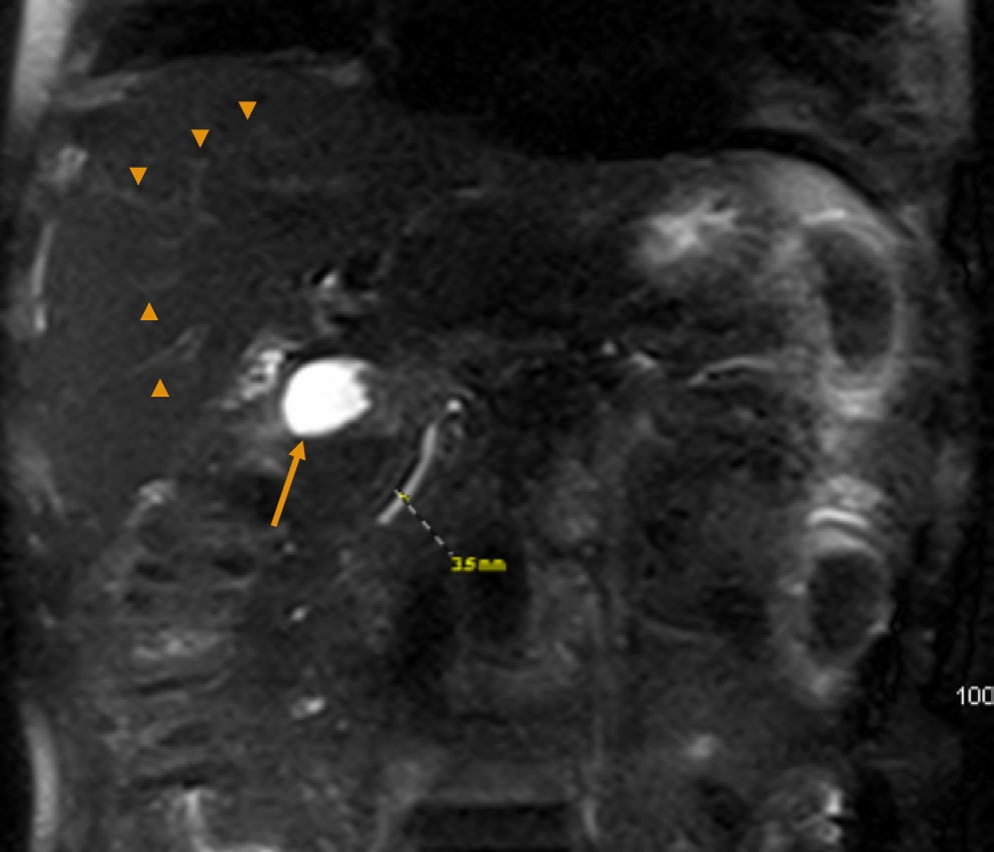
Noncontrast MRI/MRCP after admission showed normal liver parenchyma without metastatic lesions. No apparent intrahepatic ductal strictures (arrow‐heads). The gallbladder (long arrow) has multiple nonobstructing gall stones with no pericholecystic fluid, wall thickening, or ductal dilation (dashed line). No apparent hepatic hilar lymphadenopathy (not shown)

On admission, the patient was afebrile with a regular pulse at 91/min and a blood pressure of 105/47 (mean arterial pressure, MAP of 66 mm Hg). He was jaundiced, with no hepatosplenomegaly or other stigmata of liver disease. Liver function tests revealed the following: total bilirubin, 3.8 mg/dL; direct bilirubin, 3.1 mg/dL; alkaline phosphatase (ALP), 654 IU/L; gamma‐glutamyl transferase (GGT), 988 IU/L; albumin, 4 g/dL; and prothrombin time‐international normalized ratio (INR), 1.2. Serology for hepatitis B and hepatitis C infection was negative. Other admission laboratory data are summarized in Table 2. Epstein‐Barr infection and autoimmune markers were not evaluated. Abdominal ultrasonography (AUS) showed nonobstructing cholelithiasis without cholecystitis or biliary ductal dilatation, with normal portal and hepatic vasculature and no apparent lymphadenopathy. Due to the patient's kidney disease, contrast CT could not be performed. Noncontrast MRI and MRCP showed normal liver parenchyma with mild reactive hepatic hilar lymphadenopathy, three small hepatic cysts/hemangioma, and a few nonobstructing pancreatic cysts (previously described on CT during initial PCa workup) as shown in Figure 3. There was no evidence of hepatitis, liver metastases, biliary ductal dilatation or obstruction, hepatosplenomegaly, or ascites (Figure 3).

**Table 2:** Laboratory data on admission

Component	Value	Normal	Component	Value	Normal
Hematocrit	29.8%	41.0%‐53%	Albumin	4 g/dL	3.5‐5.0 g/dL
White blood cells	7.7 × 10^3^/mm^3^	4.0‐11.0 × 10^3^/mm^3^	Lactic acid	2.6 mg/dL	0.7‐2.1 mg/dL
Platelets	22.1 × 10^4^/mm^3^	150‐350 × 10^3^/mm^3^	Lipase	152 IU/L	23‐300 IU/L
AST	74 IU/L	32 IU/L	Na	135 mEq/L	137‐145 mEq/L
ALT	45 IU/L	30 IU/L	K	4.2 mEq/L	3.5‐5.1 mEq/L
Total bilirubin	3.8 mg/dL	1.2 mg/dL	CO_2_	25 mEq/L	22‐30 mEq/L
Direct bilirubin	3.1 mg/dL	0.5 mg/dL	BUN	38 mg/dL	9‐20 mg/dL
ALP	654 IU/L	335 IU/L	Creatinine	1.9 mg/dL	0.7‐1.3 mg/dL
GGT	988 IU/L	70 IU/L	Ca	10 mg//dL	8.3‐10.3 mg/dL
PT	13.1 s	10.7‐12.9 s	Mg	2.3 mEq/L	1.6‐2.3 mEq/L
INR	1.2	0.9‐1.1	Phosphorus	3.2 mEq/L	3.0‐4.5 mEq/L

Abbreviations: AST, aspartate aminotransferase; ALT, alanine aminotransferase; ALP, alkaline phosphatase; GGT, gamma‐glutamyl transferase; PT, prothrombin time; INR international normalized ratio.

Notably, serum ALP level began to decrease by day 3 of admission. Meanwhile, total bilirubin peaked at 6.5 mg/dL (direct bilirubin: 5.3 mg/dL) and trended down afterward (Figure 2). PSA was not measured during the hospitalization. However, enzalutamide therapy was discontinued on day 5 of admission given initial concerns about possible enzalutamide‐related liver injury based on laboratory abnormalities described in the Food and Drug Administration (FDA)‐approved prescribing information for enzalutamide.^9^ Consultation with oncologists deemed this possibility unlikely. The patient's clinical status deteriorated with anorexia, hemodynamic instability, and altered mental status, so the patient and his family opted for intense comfort care. Therefore, we did not pursue further diagnostic evaluation or resume enzalutamide therapy. The patient succumbed to his illness two weeks after admission.

## 3 DISCUSSION

The differential diagnoses for cholestatic jaundice in this patient broadly include hepatobiliary obstruction—from metastatic infiltration, gallstones, or pancreatic cancer^10^—and systemic etiologies like drug reactions, infections and autoimmune disorders. Very rarely, idiopathic cholestasis can be a paraneoplastic phenomenon in PCa.^2^, ^3^, ^4^, ^5^

This patient had no pre‐existing liver metastasis on contrast CT at the time of initial diagnosis of metastatic PCa (Figure 1), and his acute on chronic renal failure (ACRF) precluded contrast‐enhanced abdominal CT upon admission. Even though AUS demonstrated nonobstructive cholelithiasis, there was no associated cholecystitis, and subsequent MRI and MRCP ruled out liver metastases and other obstructive processes during this admission (Figure 3). In this elderly male with an unremarkable autoimmune predisposition and no characteristic hepatobiliary ductal strictures or dilatations, primary biliary cholangitis and sclerosing cholangitis were less likely culprits, although these etiologies are best diagnosed with serologic and histologic studies in warranted cases.^11^, ^12^, ^13^ We reliably excluded infectious and toxic etiologies based on his unremarkable viral serology and exposure history. As shown in Figure 2, the mildly elevated and rapidly normalized transaminases also ruled out ischemic hepatitis.^14^ To our knowledge, there is no known clinical evidence of impaired biliary excretion in renal disease.

The close temporal proximity between symptom onset and the initiation of enzalutamide raised suspicion about medication‐related adverse reaction. According to the original prescribing information for enzalutamide, 3% of patients taking the drug had mild‐to‐severe elevation in bilirubin compared to 2% of patients taking placebo.^9^ This data came from the AFFIRM trial, but the published report did not include statistical analysis for adverse effects.^8^ Therefore, using statistical methods described in the trial, we re‐analyzed the raw data and found no significant difference in hyperbilirubinemia between the treatment groups (data not shown). Moreover, unpublished data from the manufacturer did not show statistically significant liver toxicity even in patients with pre‐existing liver abnormalities (data not shown). In fact, the current prescribing information on enzalutamide completely excludes originally reported liver enzyme abnormalities,^15^ which we clarified through personal correspondence with the FDA (data not shown). These changes reflect favorable safety profiles demonstrated in Phase III trials, which did not show evidence of enzalutamide‐induced liver impairment.^16^, ^17^, ^18^ In addition to accruing safety data, it is possible that patient‐driven enquiries with the drug manufacturer and the FDA—as we did for our patient—may have partly prompted this change in the prescribing information..

Enzalutamide does not increase overall rates of adverse effects (AEs) compared to placebo.^6^ The most common all‐grade AEs attributed to enzalutamide include fatigue, hot flashes, and hypertension.^6^ However, both Phase III PREVAIL and AFFIRM trials demonstrated lower rates of high‐grade AEs in enzalutamide groups, and the median time to such events in placebo groups was 8‐12 months earlier than in enzalutamide groups.^7^, ^8^ Therefore, recent reviews suggest that most of these AEs were likely related to disease progression rather than the study drugs.^6^, ^19^ We also infer that incident LFT abnormalities were likely related to disease progression, and future analyses could establish this possibility. If such associations exist between cholestatic abnormalities and disease progression, without hepatobiliary involvement, they might support anecdotal reports that PnC may be more common than recognized.

Consistent with other reported cases, we presumptively diagnosed PnC upon excluding obstructive pathology and enzalutamide‐related toxicity among other common etiologies. Fatigue and cholestasis in this patient were more likely related to progression of mCRPC rather than enzalutamide. As shown in Figure 2, serum bilirubin and ALP started rising along with the precipitous progression of PSA and symptoms prior to initiation of enzalutamide therapy and hospitalization. We noted that serum ALP and bilirubin were trending down prior to discontinuing enzalutamide during the hospital course, although our patient succumbed (Figure 2). Had aggressive endocrine therapy been successful, previous case reports suggest that the cholestasis may have improved.^5^ Whereas we discontinued therapy based on the original FDA‐approved label, the PREVAIL trial, which assessed the efficacy and safety of enzalutamide in patients with similar chemotherapy‐naïve mCRPC, found that the most common fatal AEs were disease progression and general decompensation.^7^ The PREVAIL trial demonstrated a similar incidence in both study arms and no evidence of enzalutamide‐related liver impairment, even in sub‐groups with baseline liver involvement.^7^, ^17^ Clearly, cholestasis in this patient was more likely paraneoplastic than enzalutamide related.

PnC is an uncommon and a potentially underrecognized manifestation of PCa. In almost all reported cases, PnC emerged with newly established PCa or progression of known disease; it is often reversible without a known prognostic value.^2^, ^3^, ^4^, ^5^ In this case, PnC began with biochemical disease progression and coincident with initiation of enzalutamide, masquerading as a drug‐related event (Figure2). In addition to diagnostic challenges, this case presented new conundrums that underscore a systematic patient‐centered approach to clinical decision‐making and management.

Enzalutamide is broadly becoming a first‐line option with potential indications for metastatic and non‐metastatic PCa given its consistently favorable safety profile and superior survival benefits over first‐line bicalutamide.^18^ As reflected in the current FDA drug label, several studies affirm that enzalutamide does not cause clinically significant adverse liver dysfunction that warrant cessation of therapy.^15^ Still, clinicians might erroneously attribute idiopathic cholestasis or PnC to enzalutamide and cease potentially effective therapy, based on the originally inconclusive safety data in FDA‐approved drug label.^9^, ^15^PnC—is a potentially reversible and benign entity. Therefore, clinicians must weigh the therapeutic benefits of continuing enzalutamide against the unlikely chance of harmful liver injury before preemptively discontinuing therapy for idiopathic cholestasis or suspected AEs. If suspecting the later, particularly with concomitant high‐grade symptoms like fatigue—absent seizures or potentially fatal AEs—dose adjustments might be viable and effective without discontinuation.^8^

In our case, we reliably inferred that enzalutamide was an unlikely culprit upon analyzing existing literature and toxicity data with multidisciplinary input. However, we did not resume enzalutamide therapy or pursue additional diagnostic evaluation in honor of our patient's wish for comfort‐only care. Therefore, once clinicians recognize PnC or benign drug‐related AEs, they should not withhold potentially successful therapy unless to honor patients’ wishes.

## 4 CONCLUSION

Paraneoplastic cholestasis is a rare and underrecognized manifestation of PCa. It is a diagnosis of exclusion. Erroneous attribution of this entity to a medication like enzalutamide might lead to cessation of otherwise effective and beneficial therapy. Therefore, clinicians must weigh the unlikely risks of enzalutamide‐related liver injury before discontinuing therapy in idiopathic cholestasis or presumed PnC.

## CONFLICT OF INTEREST

None declared.

## AUTHOR CONTRIBUTIONS

JL, GA, and DAH: contributed to the conception and designed the study. JL and DM: contributed to direct patient care. JL and GA: collected and analyzed clinical data and drafted and revised the initial manuscript. CDT, DM, and DAH: provided critical appraisal, interpretation, and revisions for intellectual content in their respective fields of expertise. All authors read and approved the final manuscript.

## ETHICAL APPROVAL

This report was deemed a nongeneralizable nonhuman subjects research under the Health and Human Services (HHS) Protection of Human Subjects Regulations. It is also was deemed not subject to IRB oversight by our institution. Still, every effort has been made to protect the patient's identity and there is no reason to believe that our patient would have objected to this publication.

## CONSENT FOR PUBLICATION

Verbal informed consent was obtained from the deceased patient's healthcare proxy for publication of this case report and accompanying images.

## Data Availability

Data sharing is not applicable to this article as no datasets were generated or analyzed during the current study.

## References

[1] Kranidiotis GP , Voidonikola PT , Dimopoulos MK , Anastasiou‐Nana MI . Stauffer's syndrome as a prominent manifestation of renal cancer: a case report. Cases J. 2009;2(1):49. 19144140 10.1186/1757-1626-2-49 PMC2628869

[2] Bhangoo MS , Cheng B , Botta GP , Thorson P , Kosty MP . Reversible intrahepatic cholestasis in metastatic prostate cancer: an uncommon paraneoplastic syndrome. Mol Clin Oncol. 2018;8(4):613‐616. 29541472 10.3892/mco.2018.1564 PMC5838296

[3] Kato D , Okwara C , Moreland C , Parker A . Hepatic dysfunction as a paraneoplastic manifestation of metastatic prostate adenocarcinoma. J Investig Med High Impact Case Rep. 2014;2(2):2324709614539927. 10.1177/2324709614539927 PMC4528893 26425613

[4] Koruk M , Büyükberber M , Savaş C , Kadayifçi A . Paraneoplastic cholestasis associated with prostate carcinoma. Turk J Gastroenterol. 2004;15(1):53‐55. 15264123

[5] Ravindranathan D , Hitron EE , Russler GA , Xue Y , Bilen MA . Metastatic prostate cancer manifesting as cholestatic jaundice: a case report and review of the literature. Case Rep Oncol Med. 2018;2018:1809432. 29780650 10.1155/2018/1809432 PMC5892301

[6] Zhu J , Liao R , Su C , et al. Toxicity profile characteristics of novel androgen‐deprivation therapy agents in patients with prostate cancer: a meta‐analysis. Expert Rev Anticancer Ther. 2018;18(2):193‐198. 29257709 10.1080/14737140.2018.1419871

[7] Beer TM , Armstrong AJ , Rathkopf DE , et al. Enzalutamide in metastatic prostate cancer before chemotherapy. N Engl J Med. 2014;371(5):424‐433. 24881730 10.1056/NEJMoa1405095 PMC4418931

[8] Scher HI , Fizazi K , Saad F , et al. Increased survival with enzalutamide in prostate cancer after chemotherapy. N Engl J Med. 2012;367(13):1187‐1197. 22894553 10.1056/NEJMoa1207506

[9] US Food and Drug Administration . XTANDI (enzalutamide) capsules label. MD, USA: US food and drug administration; 2012:1–16. https://www.accessdata.fda.gov/drugsatfda_docs/label/2012/203415lbl.pdf. Accessed August 21, 2019.

[10] Romano G , Agrusa A , Galia M , et al. Whipple's pancreaticoduodenectomy: surgical technique and perioperative clinical outcomes in a single center. Int J Surg. 2015;21(suppl 1):S68‐71. 26122590 10.1016/j.ijsu.2015.06.062

[11] O'Connor OJ , O'Neill S , Maher MM . Imaging of biliary tract disease. Am J Roentgenol. 2011;197(4):W551‐W558. 21940525 10.2214/AJR.10.4341

[12] Khurana V , Singh T , Praveen KR , Nazer H . Primary sclerosing cholangitis: background, etiopathophysiology, epidemiology, 2017. https://emedicine.medscape.com/article/187724-overview. Accessed April 11, 2018.

[13] Pyrsopoulos NT , Reddy KR . Primary biliary cholangitis (primary biliary cirrhosis): practice essentials, background, pathophysiology, 2017. https://emedicine.medscape.com/article/171117-overview. Accessed April 11, 2018.

[14] Giannini EG , Testa R , Savarino V . Liver enzyme alteration: a guide for clinicians. CMAJ. 2005;172(3):367‐379. 15684121 10.1503/cmaj.1040752 PMC545762

[15] US Food and Drug Administration . XTANDI (Enzalutamide) Capsules Label. MD, USA: US Food and Drug Administration; 2018:1–16. https://www.accessdata.fda.gov/drugsatfda_docs/label/2018/203415s014lbl.pdf. Accessed August 21, 2019.

[16] Shore ND , Chowdhury S , Villers A , et al. Efficacy and safety of enzalutamide versus bicalutamide for patients with metastatic prostate cancer (TERRAIN): a randomised, double‐blind, phase 2 study. Lancet Oncol. 2016;17(2):153‐163. 26774508 10.1016/S1470-2045(15)00518-5

[17] Alumkal JJ , Chowdhury S , Loriot Y , et al. Effect of visceral disease site on outcomes in patients with metastatic castration‐resistant prostate cancer treated with enzalutamide in the PREVAIL trial. Clin Genitourin Cancer. 2017;15(5):610‐617.e3. 28344102 10.1016/j.clgc.2017.02.007

[18] Penson DF , Armstrong AJ , Concepcion R , et al. Enzalutamide versus bicalutamide in castration‐resistant prostate cancer: the STRIVE trial. J Clin Oncol. 2016;34(18):2098‐2106. 26811535 10.1200/JCO.2015.64.9285

[19] Golshayan AR , Antonarakis ES . Enzalutamide: an evidence‐based review of its use in the treatment of prostate cancer. Core Evid. 2013;8:27‐35. 23589709 10.2147/CE.S34747 PMC3622394

